# Distributing *Zakatu Kasbil 'Amal* as an Alternative to Student Funding, Evidence in Indonesia’s Universities

**DOI:** 10.12688/f1000research.144610.1

**Published:** 2024-03-11

**Authors:** Marliyah Marliyah, Budi Dharma, Ahmad Muhaisin B. Syarbaini

**Affiliations:** 1 Islamic Economic, Universitas Islam Negeri Sumatera Utara, North Sumatra, Indonesia; 2 Management, Universitas Islam Negeri Sumatera Utara, North Sumatra, Indonesia

**Keywords:** Improving University Quality, Income Zakat, Indonesian Universities, Student Funding.

## Abstract

**Background:**

The purpose of this paper is to clarify the use of zakat as an alternative funding for students to pay their tuition fees. Apart from that, this paper also explains how zakat collected from *muzakki* fixed income known as *zakatu kasbil 'amal*, which is known as income zakat and is still controversial in Indonesia.

**Method:**

The study uses a systematic literature review with an inductive approach to find out the theoretical basis of the research questions. The analysis uses a qualitative method that explores significant findings from various literature sources. The findings will combine with the exploration results of several universities in Indonesia that use social funds as a tool for helping students.

**Result:**

The income zakat controversy mediates using the precautionary principle, which ensures that the projected income indeed exceeds the *nisab* (1 year) of the income. Some Islamic universities in Indonesia carry out this zakat withdrawal and even use an auto debit system. On the other hand, the distribution of zakat to students is also controversial because zakat is part of worship whose distribution is regulated into nine *asnaf.* Nevertheless, students may be equated with one of the *asnaf* so that it becomes permissible to be the subject of zakat distribution. Observation findings at Indonesian Islamic universities even show that the results of zakat distribution strongly influence the success of students’ studies, even if only in aid of tuition fees.

## 1. Introduction

One of the root problems in Indonesian higher education is student funding. Providing alternative student funding will significantly contribute to higher education improvement in Indonesia. The basis for the emergence of student funding problems is the increasing costs of higher education, which stem from the university’s expectation to grow in an era of technological disruption and high competition. ^1^ Even though universities can obtain operational funding assistance from various parties, ^2^ more is needed to ease the burden transferred to students. The educational costs charged to students tend to increase yearly. ^3^

Even though Indonesian people’s participation in higher education tends to increase yearly, ^4^^,^^5^ it is dominated by communities with the highest per capita income in Indonesia. ^6^^,^^7^ BPS-Statistics Indonesia reports (2022b) show that the average cost of pursuing higher education has increased by 85.51% from the costs incurred during high school (almost two times high school cost). So, it is natural that higher education tends to be expensive.

Why is Indonesian higher education increasing its tuition fees? The research results of Marliyah & Dharma (2022) inform that the most critical risk for Indonesian Islamic universities is the risk of standardizing the higher education system, ^8^^,^^9^ which must be “ *Unggul*” (DIKTI version) and have an international ranking. Therefore, various improvements need to be made, such as improving college facilities and infrastructure and improving the quality of students. ^10^ This impact increasing university operational costs, and with the current university business model, ^11^^–^^13^ the most straightforward choice to take is to pass it on to students.

BPS-Statistics Indonesia (2022b) reports an increase in participation of the lowest economic community by 1.34 times from 2016. The main factor causing that phenomenon is funding assistance schemes, including scholarships for poor people, paving the way for students to study in universities. Popular schemes in various countries are Scholarships and endowment Funds. ^14^ In Indonesia, the Government provides higher education subsidies known as KIP ( *Kartu Indonesia Pintar*). Apart from these schemes, popular funding at various universities in Indonesia comes from social financial assistance, including zakat.

Kompas (2022), in an investigation, stated that people with middle to lower economic levels in Indonesia need assistance to obtain higher education, ^3^ and the distribution of zakat is one alternative assistance. Zakat is one of the Islamic pillars aiming to fulfil the primary needs of Muslims, one of which is gaining access to higher education. It is because Islam realizes that although all humans have the same opportunity to work and earn an income, not everyone can earn a decent and sufficient income to fulfil their life’s needs. On the other hand, zakat is an Islamic mechanism for withdrawing part of the rich’s wealth to distribute to people in need so that everyone has the same right to access and enjoy the results of the country’s development. The findings of BAZNAS Center Of Strategic Studies & Zaenal (2019) that for two consecutive years (2018-2019), ^15^ the distribution of zakat had a good impact on *mustahik* and confirmed by Abdullah et al. (2012) and Beik et al. (2017) concluded that one of the solutions to improving human quality in Indonesia is the distribution of zakat. ^16^^,^^17^

Apart from that, there is a complicated problem that requires logical and accurate research, namely whether zakat can be distributed to students. It is said to be complicated because, in Islam, the criteria or groups of people who are entitled to receive zakat have been determined in the Al Quran. In Surah At-Taubah verse 60, Allah SWT said: *"Indeed, zakat is only for the needy, the poor, the amils, those whose hearts are softened and then convert to Islam, to free those who are enslaved, to free those who are in debt, for (jihad) in the way of Allah and for those who are in a journey that requires help as an obligation from Allah. Allah is knowing and wise."*

The verse above explains that those who are entitled to receive zakat distribution are only eight groups “ *asnaf*,” namely the needy, the poor, the *amil*, Muslim converts, for freeing enslaved people, people who are in debt for mujahid ( *jihad*) defending Islam and travellers who need accommodation assistance. If we look carefully, there is no explicit information from this verse, which shows that students have the right to receive zakat distribution. It is a significant discussion that requires sufficient attention, considering that education is an essential instrument in human development, especially in Indonesia.

The researcher seeks to explore the origin of assistance from income zakat and its distribution to students for higher education purposes, both in the form of previous research findings and actual exploration in several universities in Indonesia. These findings will help universities to use zakat as alternative funding for students and provide recommendations on how to integrate them into strategic policies for the development of universities.

## 2. Theoretical framework and literature review

### 2.1 Zakat

The terminology of zakat begins with a review of its etymology, defined as *an-nama'*, which means blessing, cleanliness, and growth, or a process that will provide incredible wealth and protect it from danger. This reference is based on *Ibn Taymiyah's* translation, which states that zakat payers will have their hearts guarded to become pure. The zakat terminology is defined as an obligation from Allah to a certain amount of wealth with a specific size ( *nisab*) which must be given to those who need it/ *mustahik*, or it is called a certain level of certain wealth which must distributed to entitled people who have determined (in the Alquran) when they meet the conditions.

According to Dieb et al. (2012), *"This wealth is called zakat because the original wealth (which has been given as zakat) grows because of the blessings of zakat wealth and the prayers from the people who receive it."* Likewise, zakat is a cleanser of all wealth from *syubhat* (doubts) and a cleanser from the rights of other people attached to them. As Allah commanded, zakat is a form of worship that Muslims must carry out. It has a transcendental ( *unseen*) side and a real side, following the Islamic view of wealth that contains the rights of people experiencing poverty attached to it, then must be paid out as a form of zakat.

### 2.2 Distribution of Zakat

Zakat is one of the Islamic laws obligatory for its well-off adherents, where the goal is the same as other goals of Islamic philanthropy so that wealth does not only circulate in certain circles, as stated in the seventh verse of Surah Al-Hasyr. Zakat is an effort so that people have sufficient financial capacity to meet basic needs such as food, clothing, and shelter, not to mention the need for a decent education.

Therefore, it includes the distribution of zakat in Islamic law, which covers an extensive group, as explained in Qs. At-Taubah: 60. However, apart from the aim of meeting people’s basic needs, if we look closely at how Allah determines who is entitled to receive zakat in the Al Quran, it turns out that zakat is not only intended to meet basic needs alone. Furthermore, zakat will be distributed to those who have contributed to developing the country broadly. For example, among the *asnaf* of zakat who are not necessarily included in the poor category are *fisabilillah* and *mualaf.*

For example, *Fisabilillah* are those in the army fighting for Islam who are assigned to guard the borders and protect the Muslim territory from enemy attacks. *Fisabilillah* still gets zakat even if they are rich. ^18^ The inclusion of the *fisabilillah* group in the *asnaf* category (those who are entitled to receive zakat) is a strong indication that those who contribute to religion are entitled to receive zakat as a form of appreciation for those who are willing to serve their religion. So, among contemporary *Fiqh* (Islamic law) scholars, the *asnaf* category of zakat receives a fairly extensive portion of discourse and discussion in order to maximize the potential and influence of zakat in building an advanced and sustainable Islamic civilization.

## 3. Methods

The research seeks to explore contextual views in finding solutions to the complexity of background phenomena. On this basis, the recommended research approach is qualitative. ^19^^–^^21^ Because researchers seek to present theoretical fundamentals that can provide a comprehensive understanding of zakat in a specific context, this requires finding historical data from time to time, ^22^ which according to Fowler & Christakis (2010) must be able to be linked to broader needs, ^23^ and in line with Fisher et al. (2017) recommend a systematic literature review approach. ^24^

A systematic literature review is carried out by analysing literature related to zakat, both in the context of its collection and in the context of its distribution. The research uses the concept of inductive analysis in exploring specific evidence so that findings emerge naturally from published literature. ^25^ For this reason, the appraisal/ *'Amid* concept was developed Marliyah et al. (2023) ^26^ to reduce the data into essential findings. ^27^

The research begins with data collection from relevant literature. ^28^ Moseley et al. (2009) imply the need for a quality and structure selection process (framework) that explores the constructs of the theme variable builders. ^29^ The premise underlying the unit of analysis is a thesaurus database set formed from the results of literature searches. ^28^^,^^30^^,^^31^

Relevant literature is divided into a thesaurus database of recent findings ^32^ sourced from reputable journals (Scopus, WOS, and Sinta), applicable regulation, ^33^ and a thesaurus database originating from classical and contemporary Islamic reference books as a record of events from the previous Islamic era. Researchers build an analytical framework as follows:

1) Developing a search string ^31^ with subsequent filtering, which focuses on a “search question” to identify the database. ^28^ Collecting data as search string: collecting zakat from income or *zakatu kasbil 'amal* (زكاة كسب العمل) and distributing zakat as student funds.
2) The database is screening to potentially relevant articles by title and abstract ^32^^,^^33^ The potential paper will be carried out by selecting books or articles that focus on and contrast with the search string.
3) Eligibility and sensitivity analysis. Sensitivity analysis performs by configuring inclusion and exclusion criteria, ^25^ then the potentially relevant articles screening by inclusion and exclusion criteria. ^34^ The following criteria ( Table 1):
  Notes for Eligible articles to be screened for data abstraction. ^25^
4) Qualitative synthesis analysis was done by data abstraction and reduction using triangulation tables.

**Table 1.** Inclusion and exclusion criteria. ^25^

Inclusion criteria	Exclusion criteria
At least published in 2010 for article and current regulations. Articles whose research locus is in Islamic countries. Zakat and its impact on society.	Full text not obtained. Collecting zakat in addition to income. Distributing zakat in addition to education.

To obtain empirical findings on how universities in Indonesia use zakat, researchers conducted a deep interview and FGD (Focus Group Discussion) with philanthropic organizations formed by universities with the same aim as the research theme “using social funds as an alternative to student funding.” Sources of informants came from UPZ UIN Sumatera Utara, UPZ UIN Walisongo Semarang, Puspas Unair, and Ziswaf UNIDA. The selection (as informants) was based on the fact that they have the best image in Indonesia in collecting social funds. For example, Puspas is an organization that has long been active and plays a crucial role for Universitas Airlangga students ^35^ and managing finances very well, as reflected in the unqualified opinion acquisition for the last three years. UPZ UIN Walisongo Semarang is the best UPZ in Indonesia, according to the Ministry of Religion. ^36^ The data and information obtained will be analyzed using qualitative analysis tools such as coding analysis for data reduction and content analysis for systematically identify the meaning.

## 4. Research findings

### 4.1 Income zakat and its controversies

Income zakat is a controversial type of zakat. Until now, the legal status or position of income zakat is still being debated, which covers comprehensive aspects. If simplified, the debate can be concluded in two aspects. First, is it permissible to determine the type of zakat whose determination does not come directly from the Al Quran or Hadith? Second, if it is permitted, then what are the provisions for determining the requirements for income zakat obligations both in terms of *nisab* (minimum level) and haul (period)?

As previously explained, the zakat required in Islam, whether it comes from the Al Quran or the Hadith, is limited to six types, namely zakat on gold and silver (including) money, zakat on livestock, zakat on staple foods, zakat on trade, zakat on mining products, and the last is zakat fitrah. All types of zakat mentioned above have transparent sources and arguments in the Al Quran and Hadith.

Based on the explicit provisions related to the types of zakat mentioned above, not a single ulama since the time of the Prophet Muhammad until many later generations carried out ijtihad or reform to determine other types of zakat outside of what is stated in the Al Quran or the Hadith. It is because zakat is a *mahdhah* (ritual/form of worship) where a Muslim must obey and follow the rules without adding or subtracting from the rules. This attitude is based on the rules agreed upon by the ulama, namely:

الاصل في العباده التوقيف

“ *The original law in determining worship is to follow standard rules.*”

So based on this rule, since the zakat law was introduced by Islam, not a single Muslim researcher has modified the law, both from the type of zakat to the determination of the minimum level ( *nisab*) and the mandatory period for paying zakat ( *haul*). The zakat law is implemented following the provisions of the Al Quran and Hadith.

Discourse regarding income zakat was first raised in the 60s. The researcher who published the first work related to income zakat (professional zakat) was *Yusuf Al Qardhawi.* Through his work entitled *Fiqh Az*- *Zakah* (Fiqh Zakat) in 1969. ^37^ Al Qadhawy was the person who first made issues related to income zakat. It became widespread with challenging discussions throughout the Islamic world, both in meetings, scientific writings, and research, both pros and cons.

Even though discussions about income zakat have emerged since the late 60s, according to Riyadi (2016), the study and practice of it began to spread in Indonesia around the late 90s and early 2000s. ^38^ After Al Qardhawi’s book was translated into Indonesian by Didin Hafidhuddin with the title “Fikih Zakat” in 1999, income zakat began to be widely implemented by zakat management institutions in Indonesia, both BAZ ( *Badan Amil Zakat*) owned by the government (either BAZDA or BASNAZ), or privately owned LAZ ( *Lembaga Amil Zakat*), such as PKPU, Dompet Dhuafa.

The emergence of the idea of income zakat gave rise to widespread controversy. There are quite a few scholars who support Al Qardhawi’s ideas and even wrote many works to strengthen what he had initiated. However, there are also quite a few who oppose it and refute it with various scientific arguments for the ideas put forward by Qardhawy. In the Indonesian context, the same thing also happens. Not all fatwa institutions agree with the idea of income zakat.

*Nahdatul Ulama* (NU) in *Bahtsul Masail Maudlu'iyyah* activities at the East Java PWNU Regional Conference at Lirboyo (15-28-29 July 2018) confirmed NU’s official opinion that there is no income zakat obligation in the four *mahzab.* However, every person in any profession with money that reaches the *nisab* and *haul* must pay zakat, considering that this money has the same exchange rate as gold and silver ( *nuqud*). It differs from the Fatwa of the Indonesian Ulema Council (MUI/ *Majelis Ulama Indonesia*). MUI (2003) stated that all forms of *halal* income must be paid zakat on condition that they have reached the nisab in one year, namely 85 grams of gold. In this fatwa, what is meant by “income” is any income such as salary, honorarium, service fees, and other things that are obtained in a halal way, whether routine, such as state officials, employees, or non-routine, such as doctors, lawyers, consultants, and the like, as well as income earned from other freelance work.

However, it should be noted that even though the MUI agrees with Al Qardhawi’s ideas regarding income zakat obligations, if we look more carefully, the MUI fatwa takes a different legal perspective from several points that Al Qardhawi initiated. Among these differences is that the MUI fatwa states that all forms of *halal* income must paid zakat if they have reached the *nisab*, namely 85 grams of gold. It means that the minimum level of assets that requires income zakat is analogous ( *qiyas*) to zakat on gold and silver. It is different from Al Qardhawi’s initial idea, where the *nisab* of income zakat is debiased/analogous ( *qiyas*) to the *nisab* of agricultural zakat, namely 653 kg of dry grain or 520 kg of rice.

The MUI goes further than Al Qardhawi’s original idea, where the MUI believes that even if a person’s income has not reached the *nisab*, he can already pay zakat if, in his calculations, the amount of salary or income he receives in one year (even if it is still in the calculation above paper) and has not been received, can reach the *nisab* value without taking into account daily or monthly expenses that must be excluded from the income or salary received.

However, regardless of the debate regarding the law or the status of professional zakat itself, according to Baidowi (2018), even though the law regarding professional zakat is still controversial and not yet well known by the Muslim community in general and Muslim professional circles in the country in particular, awareness and enthusiasm to set aside a portion of income as zakat which he believes is a religious obligation that must be paid relatively high. ^39^

Moreover, the Indonesian government has issued various regulations related to zakat. However, according to Cahyani (2020), the legal position of professional zakat has not been strengthened by adequate regulations, at least in Law No. 38 of 1999 concerning Zakat Management, in Chapter IV Article 11 Paragraph 2, states the types of assets subject to zakat, and one of the types is income and services. So, indirectly, the issue of professional zakat already has rules to adhere to as a legal reference. ^40^

### 4.2 Student as Asnaf (zakat recipient)

Students are one of the groups that are still being debated whether they are one of the eight groups entitled to receive zakat. This is because, in Surah At-Taubah verse 60, no sentence explicitly indicates that students or those seeking knowledge have the right to receive zakat.

This causes not all Muslim scientists to agree that students have the right to receive zakat. However, this debate came to light when the MUI issued a fatwa in 1996 regarding providing zakat for scholarships. The fatwa states that “ *Giving zakat money for educational purposes, especially in the form of scholarships, is legal because it is included in asnaf fi sabilillah*”. ^41^

Looking at the fatwa, the main opinion is that students or people who are studying are included in the fisabilillah group or people who are fighting in the way of Allah. MUI issued this fatwa regarding the general rules in the method of interpreting the Al Quran, namely:

يبقى العموم على عمومه

“ *The general text is applied as is its generality.*”

The meaning of the rules of interpretation above is that if the text of the Al Quran and Hadith is universal without any particular meaning that limits its universality, then the text is applied universally without certain limits. In the context of Surah At-Taubah verse 60, one of the groups entitled to receive zakat mentioned by Allah SWT in that verse is *fisabilillah*, namely people who are fighting *jihad* in the way of Allah. However, the verse above does not clearly or explicitly explain what jihad means in the way of Allah.

The rules of interpretation above are: As long as the text of the Koran and Hadith is universal without any particular meaning that limits its universality, then the text is applied universally without certain limits. In the context of Surah At-Taubah verse 60, one of the groups entitled to receive zakat mentioned by Allah SWT in that verse is *fisabilillah*, namely people who are fighting jihad in the way of Allah. However, the verse above needs to clearly or explicitly explain what *jihad* means in the way of Allah.

It is true that during the time of Rasulullah SAW, the groups included in this category were war volunteers who did not have a fixed salary. ^42^ However, if viewed in general terms, jihad does not limit the scope of its meaning to troops or soldiers fighting to defend and defend the country. However, in general terms, without specifying the person in those who are fighting in the way of Allah. ^43^ So, in the logic of this fatwa, MUI sees that students or students of knowledge are people who are also fighting jihad in the way of Allah, and with their knowledge, they can defend religion and advance the nation and state so that they can include as the *fisabilillah* category.

It is just that the Indonesian Ulema Council (MUI), in its fatwa, provides strict limits or categories regarding what kind of students are entitled to receive scholarships through zakat distribution. The fatwa provides three categories that must be fulfilled by pupils or university students in order to be entitled to receive a scholarship from zakat, namely: 1) academic achievement, 2) priority for those who are less fortunate, and 3) studying knowledge that is useful for the Indonesian nation.

The Indonesian Ulema Council is not the only fatwa institution that has stated that zakat funds can distribute scholarships for students. One of the international fatwa institutions that is a reference for Muslims around the world, namely Darul Ifta' Egypt, 2007 issued a similar fatwa in fatwa sheet number 175, which contained:

يجوزُ شرعًا صرفُ الزكاة في الإنفاقِ على تدريبِ طلبة العلم، خاصَّةً إذا كانوا محتاجين، حتى إن الحنفية أجازوا نقل الزكاة من بلد إلى آخر لطالب العلم. والإنفاق على طلبة العلم يشمل تدريبهم على المهارات الضرورية؛ لأنهم يحتاجون إليها

“ *It is legally permissible to distribute zakat for scholarships for science passports, especially if they are in need. Even the Hanafi Madhab allows the transfer of zakat from one country to another for distribution to seekers of knowledge. Scholarships for students include (among other things) to train them in necessary skills because they are very shaky”* ( dar-alifta.org/ar/fatawa).

Likewise, the official fatwa institution of the Islamic State of Jordan, namely Dairah Al-Ifta, issued a similar fatwa in 2013 in fatwa sheet number 2847. However, in this case, the fatwa from *Dairah Al Ifta'* provides strict limits regarding students entitled to receive zakat. The contents of the fatwa state that: 1) He must be a student who is proven to be serious about studying so that his activities in studying cause him to be unable to work and make money. 2) The knowledge the studies are *fardhu kifayah*, whose orientation is the good of the people, both *Shar'i* knowledge, such as *fiqh* and interpretation, and general knowledge, such as medicine, economics, chemistry, and others. 3) Zakat does not come from relatives or family obliged to provide for their needs.

From the description above, contemporary ulama is more inclined to the opinion that allows zakat to be distributed through scholarships to students. Of course, this fatwa is a breath of fresh air both for the government of the Unitary State of the Republic of Indonesia and for the students themselves. For the government, this will help accelerate the human development index (HDI) ^44^ and improve the quality of human resources for the nation’s children, considering that access to higher education among young people is still very low. Alawiyah (2016) concluded that one of the leading causes is the high cost of higher education, which causes many young people of productive learning age to be unable to access it due to limited costs. ^45^^,^^46^ Quoting from official data on the Central Statistics Agency (BPS) website, it shows that the percentage of poor people in March 2022 is 9.54 percent. The number of poor people in our country in March 2022 is 26.16 million. The number of urban poor people in March 2022 was 11.82 million people, while the number of rural poor people in March 2022 was 14.34 million people.

Education is the right of all groups, from the lower middle class to the upper middle class, with supportive socio-economic conditions; of course, there will be no difficulty in reaching education up to the upper level, but this is not the case with the lower middle class, which is why there is a need for equal distribution of opportunities to obtain education. Many weak people do not continue their education to a higher level because the cost of education is expensive. ^47^

The facts of the exchange show that the ulama’s fatwa regarding the legality of distributing zakat in the form of scholarships to students was welcomed by the National Zakat Amil Agency (Baznas). Baznas distribute part of the zakat from *muzakki* (people obliged to pay zakat) through educational scholarships. Baznas even formed a particular unit to handle the distribution of zakat through student scholarships. The institution is called LBB or Baznas Scholarship Institute. LBB is a program from the Distribution and Utilization Division tasked with providing educational funds to ensure the continuity of educational programs for underprivileged/poor students as an intergenerational responsibility. ^48^

Even some of the Amil Zakat Institutions affiliated with mass organizations have also started taking steps or taking part in distributing zakat to students. Prakoso, in his research in 2022, discovered the fact that the Amil Zakat Infaq Sadaqoh Muhammadiyah Institute (LAZISMU) has distributed scholarships to students since 2016. In the 2017/2018 academic year, LAZISMU provided scholarships of IDR 818,448,000 to 508 students, while in 2021, it will provide scholarships for 6,600 students. ^49^

So, for students or prospective students who come from lower middle-class economic backgrounds, this fatwa is undoubtedly a bright hope for being able to continue higher education, provided that this fatwa can open people’s minds to distribute their zakat assets in the education sector.

### 4.3 Limitations, future research avenues, and implications

The findings might be in the light of some of its limitations, where the main limitation of this research is universities in Indonesia as a locus for seeing the impact of using zakat as a mechanism to help universities, in this case, helping students in need. The combination of Indonesian culture of helping each other with the dominance of Islamic thought has resulted in the growth of the model of using zakat in universities in Indonesia.

Future research may focus on models of collecting social funds in Islamic corridors such as *alms*/ *infaq* and even waqf. The researchers’ initial findings on the waqf model led to the emergence of a well-known university in Indonesia, namely UNIDA Gontor. It is quite an initial treatise for researching the waqf model in higher education, especially in Indonesia. Currently, both alms/infaq and waqf are still predominantly used in religious activities, although specifically for waqf, various higher education foundations have emerged that source their funds from waqf.

The research results can provide essential implications for managing Islamic-based social funds (especially zakat). The research results can be used as a reference and alternative for zakat collection in universities to build zakat collection units that focus on helping improve the quality of students, then the combination of higher education interests solely for achieving benefits, namely education for all levels of society. This finding also encourages universities to carry out zakat collection activities, which are then synchronized with the needs of universities to help students unable to complete their studies.

## Data Availability

All data underlying the results is available as part of the article and no additional source data are required.
