# Distributing *Zakatu Kasbil 'Amal* as an Alternative to Student Funding, Evidence in Indonesia’s Universities

**DOI:** 10.12688/f1000research.144610.2

**Published:** 2025-09-22

**Authors:** Marliyah Marliyah, Budi Dharma, Ahmad Muhaisin B. Syarbaini

**Affiliations:** 1 Islamic Economic, Universitas Islam Negeri Sumatera Utara, North Sumatra, Indonesia; 2 Management, Universitas Islam Negeri Sumatera Utara, North Sumatra, Indonesia

**Keywords:** Improving University Quality, Income Zakat, Indonesian Universities, Student Funding.

## Abstract

**Background:**

Rising tuition costs and unequal access to higher education have prompted Indonesian universities to explore Zakat-based funding models to support needy students. In contrast, the use of Zakat on income ( *Zakatu kasbil ‘amal*) and the *Fisabilillah* category for scholarships has been increasingly practiced; their implementation remains shaped by theological debates and institutional innovations. This study examines how universities integrate fatwa-driven legitimacy with Zakat governance to mobilize Zakat and expand student support systems.

**Method:**

This study combines a systematic literature review with an exploratory qualitative approach. Academic publications, Scopus-indexed articles, national regulations, institutional reports, and relevant fatwas were analyzed to examine how theological perspectives and institutional practices interact in mobilizing Zakat for student funding. Evidence from university cases contextualized findings, such as PUSPAS UNAIR, UPZ UIN Sumatera Utara, BAZNAS, and LAZISMU.

**Result:**

The findings reveal two applied contributions. First, Zakat on income has been institutionalized by universities through various mechanisms, including establishing *Unit Pengumpul Zakat* (UPZ), forming private Zakat Institutions or collaborating with BAZNAS and LAZISMU. Supported by Indonesian Ulema Council Fatwa No. 3/2003, these institutional models embed auto-debit payroll systems and structured governance processes, transforming Zakat on income into a sustainable funding source for student scholarships. Second, universities expand the use of the *Fisabilillah* category as a legitimate *asnaf* for distributing Zakat-based scholarships. While classical scholars traditionally limit *Fisabilillah* to battle-related jihad, contemporary fatwas, including Indonesian Ulema Council Fatwa No. 120/1996, Dar al-Ifta Egypt (2007), and Jordan’s Fatwa No. 2847 (2013), permit allocating Zakat to education when it serves the collective benefit. Universities operationalize this broader fiqh application through structured programs such as the BAZNAS Scholarship Institute, LAZISMU’s scholarship schemes, and university-managed funding initiatives.

## 1. Introduction

Inadequate student funding remains a significant challenge in Indonesian higher education. Rising tuition and living costs place a heavy burden on students, especially those from low-income households, ^1^ despite increasing participation rates. ^2^ ^,^ ^3^ Government subsidies and scholarships exist but remain insufficient to meet demand. According to Indonesia Statistics (2022), the cost of pursuing higher education is 85.51% higher than completing high school, ^4^ contributing to access being dominated by students from high-income groups, ^5^ Expanding alternative financing mechanisms is essential to improve access and promote equity in higher education.

Rising tuition is driven by university business models aimed at achieving institutional accreditation and high international rankings, ^6^ and sustaining growth amid technological disruption and global competition. ^7^ To meet these standards, universities invest heavily in infrastructure, facilities, and human capital development. ^8^ Although universities receive operational funding support from various government and private sources, ^9^ these funds are often insufficient. As a result, under existing business models, ^10^ ^–^ ^12^ rising operational costs are shifted mainly to students, leading to continuous annual increases in tuition fees. ^13^ Coupled with escalating living expenses driven by inflation and broader economic pressures, the overall cost of higher education has become increasingly prohibitive, restricting access for students from low-income households. ^14^

In the Indonesian context, the government provides higher education subsidies known as KIP ( *Kartu Indonesia Pintar*), while combining with popular fund assistance schemes, such as scholarships and endowment Funds, ^15^ these resources remain insufficient to meet the growing demand for funding access to complete higher education. ^13^ However, an underutilized funding opportunity has emerged: the growing potential of Zakat on income ( *Zakatu kasbil ‘amal* ), especially from the salaries of the university community through university-based Zakat management. In many Indonesian universities, these Zakat collections have steadily increased due to auto-debit mechanisms and stronger institutional governance. ^16^ This creates a strategic opportunity for universities to proactively establish institutional Zakat management bodies, such as UPZ ( *Unit Pengumpul Zakat*) under national Zakat boards (BAZNAS), private Zakat institution, or partnerships with Zakat institutions, to mobilize resources from within and also to integrate Zakat into sustainable student funding strategies.

The growing relevance of *Zakatu kasbil ‘amal* is supported by broader Islamic scholarship and national regulation. Following the translation and widespread adoption of Qaradawi’s Fiqh al-Zakat, ^17^ Zakat on income has gained wider acceptance and is increasingly collected through formalized mechanisms. ^18^ ^,^ ^19^ Fatwa of the Indonesian Ulema Council No. 120/MUI/II/1996 explicitly permits the allocation of Zakat for scholarships under the *Fisabilillah* category, ^20^ forming the theological basis for funding students through Zakat. To operationalize this, universities have proactively institutionalized Zakat management bodies in line with the Fatwa of the Indonesian Ulema Council No. 8/2011 on the governance of amil Zakat. ^21^ For instance, LAZISMU distributed structured scholarships, such as *Beasiswa Sang Surya*, funded primarily through Zakat on income collected from lecturers, staff, and public contributors. ^22^ Similarly, PUSPAS UNAIR integrates Zakat, infaq, and sadaqah into tuition support, becoming a model for university-based social funding. ^23^ At the national level, the BAZNAS Scholarship Institute (LBB) channels Zakat to support students, ^24^ to improve human quality in Indonesia. ^25^

Despite the growing potential of *Zakatu kasbil ‘amal* as an alternative funding source for higher education, significant gaps remain in both theory and practice. There are unresolved theological debates over interpreting the *Fisabilillah* category regarding student funding. Classical scholars adopt a narrow view, limiting Zakat under *Fisabilillah* primarily to battlefield jihad and excluding students unless they qualify as faqir or poor. ^26^ ^,^ ^27^ In contrast, contemporary scholars such as al-Qaradawi and the Indonesian Ulema Council embrace a broader interpretation, ^20^ permitting Zakat-funded scholarships for students pursuing knowledge beneficial to society, even when not explicitly linked to Islamic da’wah or extreme poverty. ^28^ While universities establish Zakat management bodies and increasingly collect Zakat on income, their approaches to managing and distributing these funds remain fragmented and inconsistent. Some institutions, like BAZNAS, LAZISMU, and PUSPAS UNAIR, have operationalized the broad interpretation by providing structured scholarships, while others lack clear frameworks or eligibility criteria.

Existing research predominantly focuses on theological justifications and normative debates, ^29^ ^,^ ^30^ offering little insight into how universities operationalize these interpretations institutionally or evaluate their impact on educational equity. This creates a critical knowledge gap: despite a clear regulatory foundation and ongoing scholarly debates, there is limited understanding of how universities interpret, institutionalize, and implement Zakat-based student funding models within Indonesia’s higher education system. Therefore, this study aims to explore the role of Zakat on income ( *Zakatu kasbil ‘amal* ) as an alternative source of student funding in Indonesian universities. It systematically reviews the literature on Zakat-based scholarships and integrates institutional examples from selected universities. The findings provide insights into how Zakat funds are allocated to students under the *Fisabilillah* category and highlight the strategic role of universities in institutionalizing Zakat management frameworks to enhance the effectiveness of Zakat-based student funding models.

## 2. Theoretical framework and literature review

### 2.1 Zakat and its contemporary relevance

Zakat, derived from the root word *an-namā’*, signifies blessing, growth, and purification. In Islamic jurisprudence, it refers to the obligatory allocation of a portion of wealth (nisab) to those entitled under the Al-Qur’an (QS. At-Tawbah: 60). As Ibn Taymiyyah explains, Zakat not only redistributes wealth but also purifies the heart of the payer. ^31^ Similarly, Djeb et al. (2012) highlight that Zakat *“cleanses wealth from doubts (subuhat) and protects society’s collective rights.*” ^32^

In contemporary contexts, Zakat has evolved beyond personal obligation to become a strategic financial instrument for reducing inequality and supporting human capital development. ^33^ Particularly, Zakat on income ( *Zakatu kasbil ‘amal* ) has gained prominence following al-Qaradawi’s argument that fixed salaries qualify for Zakat under modern conditions. ^17^ ^,^ ^34^ This shift has expanded Zakat’s potential to fund broader social objectives, including education and poverty alleviation. ^35^

### 2.2 Zakat Distribution as Student Funding *Fisabilillah* Category

One of the most dynamic debates in contemporary Zakat scholarship concerns the scope of the Fisabilillah category, especially regarding Zakat-funded student scholarships.

**Table 1.** Debates on *Fisabilillah* and Student Funding.

Perspective	Definition of *Fisabilillah*	Scholars/Sources	Implications for Student Funding
Classical Restrictive View	Refers narrowly to battlefield jihad and direct military activity. Students are not automatically eligible unless categorized as faqir or poor.	Az-Zuhaili (2006); Sabiq (2008). ^26^ ^,^ ^27^	Scholarships are allowed only if students meet poverty criteria.
Middle View	Broadens *Fisabilillah* to include jihad of knowledge and da’wah. Students pursuing religious or societal-benefit knowledge may qualify.	Yusuf al-Qaradawi; Kahf; Chapra. ^17^ ^,^ ^36^ ^,^ ^37^	Students included conditionally under beneficial knowledge criteria.
Contemporary Broad View	Defines *Fisabilillah* as all efforts serving Islam and public welfare, including education. Explicitly adopted by Fatwa No.120/MUI/II/1996.	Indonesian Ulema Council; Widiastuti et al., 2018; Ramadhani & Hamzah, 2024. ^20^ ^,^ ^28^ ^,^ ^33^	Zakat-based scholarships are widely accepted, even for students outside Islamic fields, if they benefit society.

In Indonesia, the Fatwa No.120/MUI/II/1996 is the normative foundation allowing Zakat allocation for educational purposes, explicitly including scholarships under the *Fisabilillah* category. However, classical scholars like az-Zuhaili and Sabiq remain cautious, arguing that students should only receive Zakat if they are poor. These unresolved debates create ambiguity in how Zakat for education is operationalized.

### 2.3 Institutional Practices of Zakat-Based Scholarships

Several Indonesian institutions have adopted the broader interpretation in response to these debates. BAZNAS (National Zakat Board) operates the *Lembaga Beasiswa* BAZNAS (LBB), funding thousands of students nationwide using Zakat under *Fisabilillah.* ^24^ LAZISMU runs the *Beasiswa Sang Surya* program, directly channeling Zakat on income from lecturers, staff, and public contributors to students. ^22^ PUSPAS UNAIR integrates Zakat, infaq, and sadaqah into tuition support, recognized as a model for university-based social funding. ^23^ Several universities have established *Unit Pengumpul Zakat* (UPZ) under BAZNAS, institutionalizing Zakat collection and scholarship distribution. ^6^ However, **implementation remains fragmented**, as **Eligibility criteria** differ across institutions. Some prioritize **faqir/poor** status; others adopt **beneficial knowledge** as the main condition. Coordination between universities and national Zakat bodies remains inconsistent.

### 2.4 Universities as Institutional Actors in Zakat Management

**Figure 1.**
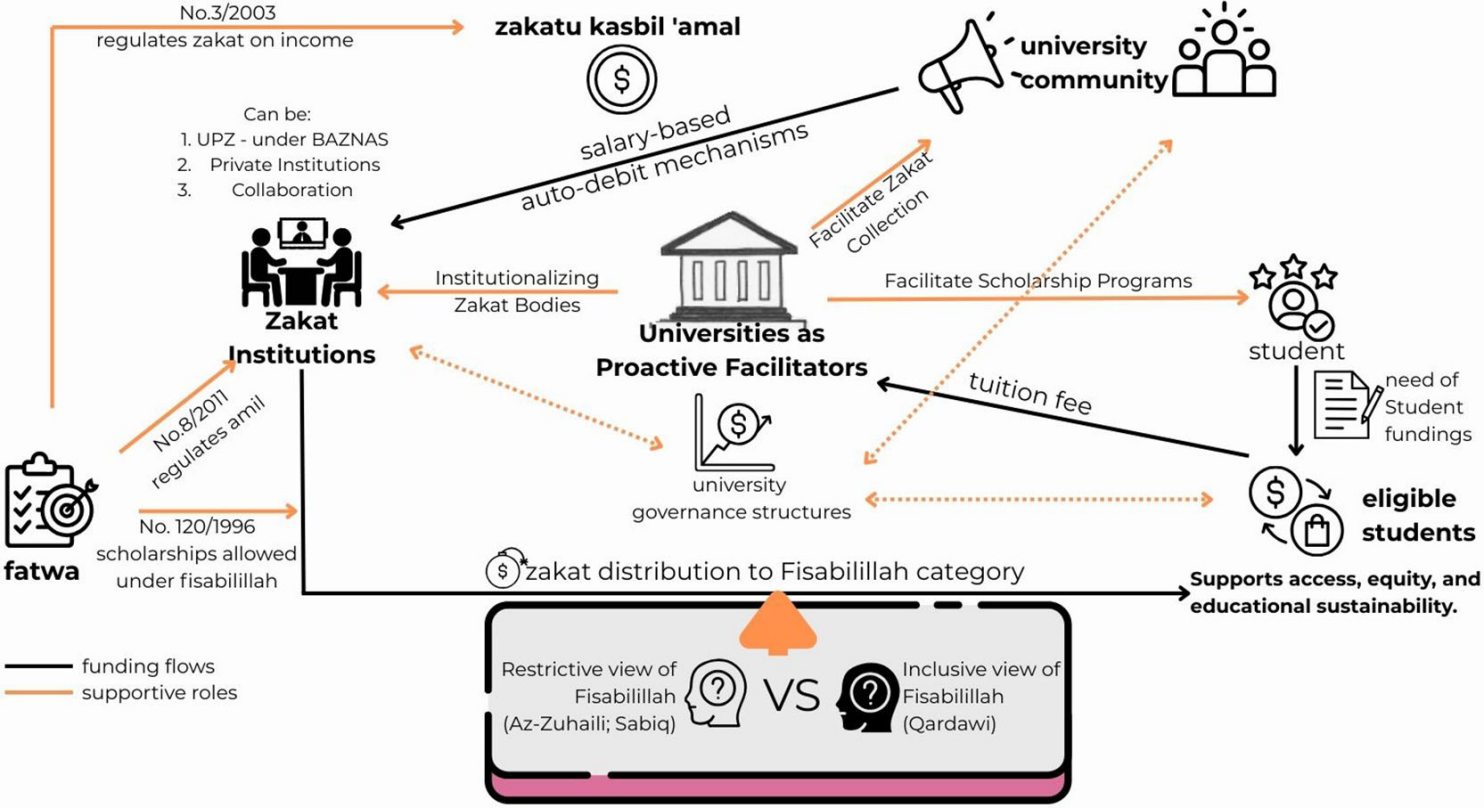
Conceptual Framework: Universities as Proactive Facilitators in Zakat Governance.

In this study, universities are conceptualized as **proactive facilitators (** Figure 1 **)** that institutionalize Zakat governance, mobilize internal resources, and enable Zakat bodies to channel Zakat on income effectively for student funding. Building on Fatwa No.120/MUI/II/1996, which explicitly permits Zakat allocation for scholarships under *Fisabilillah*, and Fatwa No.8/2011, which governs the formation of **amil Zakat** institutions. Universities **do not act as an amil Zakat** but instead **enable Zakat bodies** within their institutions to operate effectively.

The university’s strategic role involves two interrelated dimensions: First, it institutionalizes Zakat bodies, like establishing *Unit Pengumpul Zakat* (UPZ) or similar bodies under BAZNAS’ legitimacy. Or, collaborating with national (e.g., BAZNAS) and private Zakat institutions (e.g., LAZISMU). Then, integrating Zakat management into university governance structures. ^6^ Universities act as institutional actors by leveraging salary-based auto-debit mechanisms and transparent governance frameworks. Universities facilitate efficient Zakat collection from the university community, while promoting compliance through institutional campaigns and governance frameworks that encourage participation. ^22^ ^,^ ^38^ Beyond collection, universities also act as trust anchors within academic communities, ensuring that Zakat participation rates are strengthened through transparent systems and accountability mechanisms.

In parallel, universities increasingly facilitate Zakat-based student funding by supporting Zakat management bodies within their institutions. While allocation decisions remain under certified Zakat institutions, universities promote scholarship programs, identify eligible students, and support Zakat bodies in managing Zakat-based scholarships. This approach operationalizes Fatwa No. 120/1996, translating theological permissions into practical mechanisms for helping students under *Fisabilillah.* ^6^ In doing so, universities bridge restrictive interpretations, which limit student funding to those classified as faqir or poor, ^26^ ^,^ ^27^ with inclusive views that consider students pursuing beneficial knowledge as eligible under *Fisabilillah.* ^17^ ^,^ ^28^

## 3. Methods

This study adopts an interpretivist paradigm, recognizing that the meanings of Zakat on income ( *Zakat kasbil ‘amal* ) and Zakat distribution under the *Fisabilillah* category are socially and contextually constructed. ^39^ The aim is not to establish universal causal laws but to understand how universities act as proactive facilitators in mobilizing Zakat on income and channeling it effectively to support students. ^40^ An abductive reasoning approach underpins this study, allowing iterative movement between theory and empirical evidence. ^41^ ^,^ ^42^ The process begins with existing theoretical perspectives, from classical and contemporary interpretations of Zakat, regulatory fatwas, and scholarly debates on *Fisabilillah*, and integrates institutional insights from Zakat bodies in universities. Through this approach, the study develops a new conceptual framework and validates it using real-world institutional practices. ^43^

This study employs a systematic literature review (SLR) as the primary research strategy to synthesize current knowledge ^44^ and uncover insights ^45^ on How Zakat on income is mobilized and managed within universities, how Zakat is distributed under the *Fisabilillah* category to support student funding, and the strategic role of universities in institutionalizing Zakat governance through Zakat bodies. ^46^ While the SLR forms the foundation of the study, it is complemented by a qualitative case-based approach. Reports and documents from institutional Zakat bodies are used to justify and validate the emerging conceptual framework. This hybrid design ensures that the study integrates theoretical synthesis and practical grounding.

Four categories of data inform the study: academic literature, peer-reviewed articles, books, and conference papers obtained from Scopus, Web of Science, Sinta, and Google Scholar. Second is Islamic jurisprudence, which includes classical interpretations and contemporary perspectives. Third is the regulatory frameworks in the Indonesian context. Last, the institutional reports and documentation of various Zakat bodies within Indonesian universities illustrate real-world practices of mobilizing Zakat on income and distributing scholarships.

The research began with a systematic literature review to synthesize relevant findings on Zakat on income ( *Zakat kasbil ‘amal* ), Zakat-based scholarships, and the role of universities in institutional Zakat governance. The review followed established protocols to ensure methodological rigor and transparency. ^47^ ^–^ ^49^

1) The literature search was conducted across reputable databases using a predefined search string. ^49^ Keywords: combined terms related to: ^47^
Zakat on income/ *Zakatu kasbil ‘amal* ( زكاة كسب العمل ): *“Zakat kasbil ‘amal”* OR *“income Zakat”*,
Student funding and scholarships: *“Fisabilillah”* OR *“Zakat scholarship”* OR *“Zakat-based student funding”*,
University governance: *“university Zakat management”* OR *“UPZ”.*
2) After removing duplicates, the retrieved articles were screened by title and abstract to assess their relevance to the research objectives. ^50^ ^,^ ^51^ Potentially eligible studies were then assessed in full text based on clearly defined inclusion criteria ^44^ and exclusion criteria: ^52^
3) The final dataset included academic studies, fatwas, regulatory frameworks, and institutional reports. These findings were then synthesized using qualitative content analysis to build an analytical framework integrating theological debates, regulatory fatwas, and institutional practices.

**Table 2.** Inclusion and exclusion criteria. ^25^

Inclusion criteria	Exclusion criteria
Published after 2010. Research focusing on Zakat kasbil ‘amal, Zakat distribution under Fisabilillah, Zakat-based scholarships, or university-based Zakat governance. Regulatory documents, fatwas, and institutional reports relevant to the study.	Studies without full-text availability. Research unrelated to Zakat mobilization or student funding. Papers addressing general Zakat practices without a connection to education or universities.

The analysis was conducted in three iterative stages:

1) SLR Synthesis was used to identify key patterns in scholarly debates, fatwas, and Zakat governance models. The findings formed the theoretical foundation for developing a new conceptual framework.
2) Framework Development, through abductive reasoning, insights from literature were integrated with institutional perspectives to construct a framework positioning universities as proactive facilitators in mobilizing Zakat on income and distributing scholarships under *Fisabilillah.*
3) Case-Based Justification using institutional documents and reports was analyzed to validate key propositions of the framework. These real-world examples illustrate how universities implement auto-debit systems, manage Zakat bodies, and collaborate with national Zakat institutions to support students effectively.
4) Sources of case-based data came from UIN Sumatera Utara, Airlangga University. The selection was based on the fact that they have the best image in Indonesia in collecting Zakat as student funding. For example, Airlangga University with PUSPAS UNAIR is an organization that has long been active and plays a crucial role for Airlangga University students ^53^ and manages finances well, as reflected in the unqualified opinion acquisition for the last three years.

To ensure rigor and credibility, the study applied systematic review protocols ^46^ to guide the SLR. Triangulated findings from literature, fatwas, and institutional documents to enhance validity. ^54^ Used thick descriptions of institutional cases to improve transferability. ^39^ ^,^ ^54^ Followed a transparent reporting process aligned with PRISMA standards. ^56^ Combining a systematic literature review with a qualitative case-based justification, this study develops a new conceptual framework that integrates theological debates, regulatory fatwas, and institutional practices. The methodology ensures that the proposed framework is theoretically grounded and practically validated, highlighting the dual role of universities in mobilizing Zakat on income and distributing Zakat effectively under the *Fisabilillah* category.

## 4. Research findings

### 4.1 Legitimacy of *Zakat Kasbil ‘Amal*

The emergence of **Zakat on income** ( *Zakat kasbil ‘amal* ) has become one of the most dynamic themes in contemporary Zakat scholarship. Across **28 studies reviewed** (2003–2024), the following major clusters emerge:

1) Theological Legitimacy and Juristic Debates.

In classical *fiqh*, Zakat obligations applied to visible wealth ( *amwāl* ), including agricultural produce, livestock, trade commodities, and gold or silver savings. According to Ibn Qudāmah, al-Mughnī and al-Nawawī, al-Majmūʿ, salaries and wages were excluded unless accumulated, exceeded the *nisab*, and remained untouched for one lunar year ( *ḥawl* ) (Ibn Qudāmah, al-Mughnī; al-Nawawī, al-Majmūʿ). Az-Zuhaili supports this restrictive view, arguing that employment income lacked precedent during early Islamic economies. ^27^

However, the rise of modern wage-based economies prompted contemporary scholars to revisit these rulings. Qaradawi’s seminal work Fiqh al-Zakat (1969; 2020 ed.) marked a turning point, ^17^ extending Zakat obligations to recurring salaries by *qiyās* (analogical reasoning) with trade profits ( *Zakat tijarah*). Qaradawi further appeals to *tazkiyah* (purification) and *maslahah mursalah* (public interest), framing Zakat on income as a necessary mechanism for equitable wealth redistribution in modern contexts. Similarly, Mahmud and Haneef (2008) highlight how contemporary fatwas use ijtihad to reconcile classical frameworks with evolving socio-economic realities. ^57^ Despite growing acceptance, scholarly contestation remains active. Some fiqh scholars still warn against overextending Zakat categories without a clear textual basis. These tensions underscore the duality between maintaining scriptural fidelity and responding to economic transformation.

2) Behavioral Compliance and Awareness Gaps.

As juristic consensus evolved, research shifted toward behavioral compliance and determinants influencing *Zakat kasbil ’amal.* Studies rooted in the Theory of Reasoned Action by Bidin, Idris & Shamsudin (2009) highlight attitude and subjective norms as the strongest predictors of compliance. ^58^ Similarly, Bidin and Md Idris (2020) find that perceived religiosity and peer influence significantly shape willingness to contribute. ^59^ Despite widespread institutionalization, awareness gaps persist. Wahid, Ahmad and Noor (2007) reveal that many eligible Zakat payers either lack sufficient knowledge or mistrust Zakat institutions. ^60^ Later studies emphasize the centrality of trust and transparency: Saad and Haniffa (2014) reveal that credible, well-governed institutions correlate strongly with compliance levels, ^61^ leading to the possibility of operating *Zakat kasbil ‘amal* in universities.

3) Institutional Transformation and Indonesian Context.

The debates around Zakat on income have also influenced institutional reforms. Empirical evidence from a notable study, Andam & Osman (2019), reinforces these insights, showing that salaried Muslims’ intention to pay income Zakat correlates positively with institutional trust, religious commitment, and social norms. This finding validates broader behavioral patterns identified across Southeast Asia while confirming that institutional strength is pivotal to successful implementation. ^18^ In Indonesia, these shifts were codified through the Indonesian Ulema Council’s No 3/2003, according to Zakat on income ^62^ and institutionalized into national policy by Law No. 23/2011 on Zakat management, empowering BAZNAS and licensed Zakat institutions (LAZ) to collect Zakat on income systematically.

4) Emerging Research Trends.

Bibliometric reviews from Johari et al. (2014) and Alshater et al. (2021) identify income Zakat as an increasingly visible research theme within Islamic finance. ^29^ ^,^ ^30^ Emerging directions include digital Zakat platforms and fintech integration for automated collection. New Zakat sources tied to gig economy earnings and technology-based income streams. Aligning Zakat on income with productive Zakat models and broader objectives, particularly poverty alleviation and education.

*Zakat kasbil ‘amal* has evolved from a marginal juristic debate into a cornerstone of modern Zakat governance. While classical scholars remain cautious, contemporary interpretations, supported by empirical studies and institutional reforms, have enabled Zakat on income to become a strategic fiscal mechanism, underpinning initiatives such as Zakat-funded scholarships and empowerment programs.

### 4.2 Institutional Mobilization of *Zakat Kasbil ‘Amal*

The second theme examines how universities and other institutions operationalize Zakat governance to effectively mobilize *Zakat kasbil ‘amal.* Findings from peer-reviewed studies and institutional reports reveal a hybrid governance model where universities act as facilitators, leveraging their organizational structures to enhance Zakat collection, distribution, and accountability. Consistent with Wahab & Rahman (2012) and Aziz et al. (2021), at least three interrelated mechanisms underpin effective mobilization. ^63^ ^–^ ^65^ digital integration and payroll-based auto-debit systems, ^22^ institutional campaigns and awareness-building within academic communities, ^38^ and collaboration with certified Zakat bodies to ensure compliance with regulatory frameworks. ^66^

These findings align with case studies from institutional reports, such as the PUSPAS model at Universitas Airlangga, where Zakat, infaq, and sadaqah contributions are integrated to sustain scholarship funding. ^23^ ^,^ ^53^ Similarly, UPZ units under BAZNAS have formalized Zakat collection within universities, ^67^ illustrating how institutional governance frameworks mobilize significant internal Zakat resources while maintaining transparency and compliance. ^68^ ^,^ ^69^

### 4.3 Institutional Mobilization of *Zakat Kasbil ‘Amal*

The third theme examines the evolving legitimacy of the *Fisabilillah* category in contemporary Zakat governance, particularly its application to education and scholarship funding. Findings combined with fatwas, institutional reports, and peer-reviewed studies reveal a paradigm shift from a restrictive interpretation limited to combatants toward a broader, *maqaṣid*-driven framework that integrates education as a strategic component of Zakat distribution.

Classical interpretations of *Fisabilillah* were restrictive, traditionally referring to military expeditions or defending Islam’s territorial and religious integrity. Under this view, students seeking knowledge were not automatically eligible unless categorized as *faqīr* (destitute) or poor. This interpretation is reflected in Ibn Qudamah (al-Mughnī), associating *Fisabilillah* exclusively with combatants. Sayyid Sabiq (in Fiqh al-Sunnah, 2015), emphasizing its martial scope. ^26^ Wahbah az-Zuhaili (in Fiqh al-Islām wa Adillatuh, 2011) warns against diluting Zakat’s priorities. ^27^

However, contemporary scholars advocate a broader, *maqaṣid*-oriented interpretation. Yusuf al-Qaradawi argues that seeking beneficial knowledge in the service of the ummah qualifies as striving in Allah’s cause and thus falls under *Fisabilillah.* ^17^ Supporting this, Ramadhani & Hamzah (2024) emphasize that funding higher education advances human capital development, which strengthens collective welfare. ^28^ This shift reflects a growing tendency to integrate Zakat distribution with broader socio-economic objectives, aligning religious obligations with modern developmental needs. Fatwas play a central role in operationalizing the broader interpretation of *Fisabilillah **.*** Indonesian Ulema Council Fatwa No. 120/MUI/II/1996 explicitly permits allocating Zakat to scholarships for students, interpreting the Quranic reference to *Fisabilillah* inclusively:

“ *Giving Zakat money for educational purposes, especially in the form of scholarships, is legal because it is included in the asnāf fī sabīlillāh.*”

Darul Ifta, Egypt (2007) issued Fatwa No. 175, allowing Zakat to fund skills training and educational scholarships for seekers of knowledge, even permitting cross-border allocations. ^70^ Dairah al-Ifta, Jordan (2013) followed a similar path through Fatwa No. 2847, stipulating specific eligibility criteria, like: students must demonstrate academic seriousness, studies must benefit society (e.g., medicine, economics, chemistry), and funding excludes those whose families are already obliged to provide for them. ^71^ Together, these fatwas create a transnational consensus legitimizing Zakat-funded education, with the Indonesian Ulema Council framework setting a significant precedent for Southeast Asia. Despite growing consensus, debates persist:

**Table 3.** Debate in *Fisabilillah* Category.

Debate Theme	Restrictive View	Inclusive View
Scope	Limited to defending Islam (Ibn Qudamah, Sabiq)	Includes education and human capital development (Qaradawi)
Eligibility Criteria	Only faqīr or miskīn students qualify	Open to merit- and purpose-based scholarships
Priority Concerns	Risks of diverting Zakat from primary groups	Expands Zakat’s socio-economic impact sustainably

### 4.4 Operationalizing Student Funding under *Fisabilillah*

The fourth theme focuses on how Zakat funds collected within universities are allocated to students under the *Fisabilillah* category, operationalizing fatwas into practical scholarship mechanisms. Institutional reports show how fatwa-driven legitimacy translates into practice:

1) BAZNAS has institutionalized Zakat-funded scholarships via the *Lembaga Beasiswa BAZNAS* (LBB), a specialized division within its Distribution and Utilization Directorate. LBB ensures continuity of educational support as an intergenerational responsibility. ^72^
2) LAZISMU (affiliated with Muhammadiyah) has actively distributed Zakat-funded scholarships since 2016. Between 2017 and 2021, LAZISMU allocated over IDR 818 billion to thousands of students. ^22^
3) Universities increasingly establish *Unit Pengumpul Zakat* (UPZ) and funding bodies like PUSPAS UNAIR to collect Zakat from lecturers and staff. These mechanisms integrate salary-based auto-debit systems and infaq allocations to sustain scholarship funds. ^6^ ^,^ ^23^

These practices position universities and Zakat institutions as facilitators of social welfare by mobilizing Zakat on income toward human capital development. Peer-reviewed studies validate these institutional practices, like Kuanova et al (2021) findings that Zakat under *Fisabilillah* are increasingly applied beyond classical interpretations, funding education, disaster response, and socio-economic empowerment, aligning with the global development goals. ^73^ It highlights proactive Zakat boards funding modern welfare initiatives. On the other hand, Johari et al (2014) discuss the role of Zakat under categories such as *Fisabilillah*, which have seen different institutional interpretations. While some authorities restrict it to classical purposes, others allocate funds to education, skills training, scholarships, and community development. ^30^

While these initiatives reflect increasing institutional engagement, practices remain fragmented across Indonesian universities. Differences in fatwa interpretations, institutional capacity, and collaboration models create variations in scholarship eligibility criteria and fund allocation frameworks. This reveals a systemic gap: although Zakat on income collections within universities are rising, there is limited systematic evidence on how these funds are integrated into sustainable student funding strategies. This finding highlights the need for stronger governance frameworks and standardized mechanisms to realize Zakat’s potential fully.

Table 4 summarizes how universities and Zakat institutions operationalize Zakat on income to support student scholarships under the *Fisabilillah* category. The data shows institutional diversity in collection mechanisms, ranging from auto-debit payroll deductions at UPZ distribution at UIN Sumatera Utara, centralized at PUSPAS UNAIR, scholarship schemes through LAZISMU, and nationwide grants managed by the BAZNAS Scholarship Institute. These models reflect an increasing institutional capacity to mobilize Zakat resources for educational purposes, reinforcing the shift toward a *maqāṣid*-aligned understanding of *Fisabilillah* that prioritizes human capital development.

**Table 4.** Mechanisms of Zakat on Income Mobilization (in 2021 document).

Institution	Zakat Collected (Rp)	Students Funded	Distribution Mechanism	Key Insights
UPZ UIN Sumatera Utara	~Rp 2,222 billion	1,121 students	Centralized distribution	UPZ integrates payroll deductions with Zakat-based scholarships.
PUSPAS UNAIR	~Rp 172,25 million	145 students	Auto-debit, direct transfer	channels Zakat into scholarships via campus collection models.
LAZISMU	Rp 818m (2017/18) to Rp 6.6b (2021)	6,600 students	Scholarship scheme	Continuous scaling of Zakat-based scholarships since 2016.
BAZNAS Scholarship Institute	~Rp 14.118 trillion	1,670 students	Nationwide grants	Implements intergenerational funding for nationwide reach (BAZNAS and *Kader Ulama* Scholarships).

## 5. Discussion

### 5.1 Interpreting the Findings: The Ongoing Debate on *Zakat Kasbil ‘Amal*

*Zakat kasbil ‘amal* is a controversial type of Zakat. Until now, the legal status or position of income Zakat has been debated, covering comprehensive aspects. If simplified, the debate can be concluded in two aspects. First, is it permissible to determine the type of Zakat whose determination does not come directly from the Al-Qur’an or Hadith? Second, if permitted, what are the provisions for determining the requirements for income Zakat obligations in terms of *nisab* (minimum level) and *haul* (period)?

Discourse regarding income Zakat was first raised in the 60s. The researcher who published the first work related to income Zakat (professional Zakat) was *Qaradawi.* Through his work entitled *Fiqh Az*- *Zakah* (Fiqh Zakat) in 1969. ^17^ Qaradawi was the person who first raised issues related to income Zakat. Even though discussions about income Zakat have emerged since the late 60s, according to Riyadi (2016), the study and practice of it began to spread in Indonesia around the late 90s and early 2000s. ^74^

*Nahdatul Ulama* (NU) in *Bahtsul Masail Maudlu’iyyah* activities at the East Java PWNU Regional Conference at Lirboyo (15-28-29 July 2018) confirmed NU’s official opinion that there is no income Zakat obligation in the four *mahzab.* However, every person in any profession with money that reaches the *nisab* and *haul* must pay Zakat, considering that this money has the exact exchange rate as gold and silver ( *nuqud*). It differs from the Fatwa of the Indonesian Ulema Council. It stated that all *halal* income must be paid Zakat to reach the *nisab* in one year, namely 85 grams of gold. In this fatwa, what is meant by “ *income*” is any income, such as salary, honorarium, service fees, and other things that are obtained in a halal way, whether routine, such as state officials, employees, or non-routine, such as doctors, lawyers, consultants, and the like, as well as income earned from other freelance work.

However, it should be noted that even though the Indonesian Ulema Council agrees with Qaradawi’s ideas regarding income Zakat obligations, if we look more carefully, the Indonesian Ulema Council’s fatwa takes a different legal perspective from several points that Qaradawi initiated. Among these differences is that the Indonesian Ulema Council fatwa states that all *halal* income must pay Zakat if they have reached the *nisab*, namely 85 grams of gold. This means that Zakat’s minimum level of assets that require income is analogous ( *qiyas*) to Zakat in gold and silver. It is different from Qaradawi’s initial idea, where the *nisab* of income Zakat is debiased/analogous ( *qiyas*) to the *nisab* of agricultural Zakat, namely 653 kg of dry grain or 520 kg of rice.

The Indonesian Ulema Council goes further than Qaradawi’s original idea, where they believes that even if a person’s income has not reached the *nisab*, muslim can already pay Zakat if, in his calculations, the amount of salary or income he receives in one year (even if it is still in the calculation above paper) and has not been received, can reach the *nisab* value without taking into account daily or monthly expenses that must be excluded from the income or salary received. However, regardless of the debate regarding the law or the status of professional Zakat itself, according to Baidowi (2018), even though the law regarding professional Zakat is still controversial and not yet well known by the Muslim community in general and Muslim professional circles in the country in particular, awareness and enthusiasm to set aside a portion of income as Zakat which he believes is a religious obligation that must be paid relatively high. ^75^

Moreover, the Indonesian government has issued various regulations related to Zakat. However, according to Cahyani (2020), the legal position of professional Zakat has not been strengthened by adequate regulations, at least in Law No. 38 of 1999 concerning Zakat Management, in Chapter IV Article 11 Paragraph 2, which states the types of assets subject to Zakat, and one of the types is income and services. So, indirectly, the issue of professional Zakat already has rules to adhere to as a legal reference. ^76^

### 5.2 Interpreting the Findings: The Ongoing Debate on Fisabillah in the Asnaf Category

Looking at the fatwa, the main opinion is that students or people who are studying are included in the *Fisabilillah* group or people who are fighting in the way of Allah. The Indonesian Ulema Council issued this fatwa regarding the general rules in the method of interpreting the Al-Qur’an, namely:

يبقى العموم على عمومه

“ *The general text is applied as is its generality.*”

The meaning of the rules of interpretation above is that if the text of the Al-Qur’an and Hadith is universal without any particular meaning that limits its universality, then the text is applied universally without certain limits. In the context of Surah At-Taubah verse 60, one of the groups entitled to receive Zakat mentioned by Allah SWT is *Fisabilillah*, namely, people fighting *jihad* in the way of Allah. However, the verse above does not clearly or explicitly explain what jihad means in the way of Allah.

The rules of interpretation above are as follows: As long as the text of the Al-Qur’an and Hadith is universal without any particular meaning that limits its universality, then the text is applied universally without certain limits. In the context of Surah At-Taubah verse 60, one of the groups entitled to receive Zakat mentioned by Allah SWT is *Fisabilillah*, namely, people fighting jihad in the way of Allah. However, the verse above needs to clearly or explicitly explain what *jihad* means in the way of Allah.

It is true that during the time of Rasulullah SAW, the groups included in this category were war volunteers who did not have a fixed salary. ^77^ However, if viewed in general terms, jihad does not limit the scope of its meaning to troops or soldiers fighting to defend the country. However, in general terms, without specifying the person among those who are fighting in the way of Allah. ^78^ So, in the logic of this fatwa, the Indonesian Ulema Council sees that students or students of knowledge are people who are also fighting jihad in the way of Allah, and with their knowledge, they can defend religion and advance the nation and state so that they can be included in the *Fisabilillah* category.

It is just that the Indonesian Ulema Council, in its fatwa, provides strict limits or categories regarding what kind of students are entitled to receive scholarships through Zakat distribution. The fatwa provides three categories that must be fulfilled by pupils or university students to be entitled to receive a scholarship from Zakat, namely: 1) academic achievement, 2) priority for those who are less fortunate, and 3) studying knowledge that is useful for the Indonesian nation. The Indonesian Ulema Council is not the only fatwa institution that has stated that Zakat funds can distribute scholarships for students. One of the international fatwa institutions that is a reference for Muslims around the world, namely Darul Ifta’ Egypt, 2007 issued a similar fatwa in fatwa sheet number 175, which contained:

يجوزُ شرعًا صرفُ الزكاة في الإنفاقِ على تدريبِ طلبة العلم، خاصَّةً إذا كانوا محتاجين، حتى إن الحنفية أجازوا نقل الزكاة من بلد إلى آخر لطالب العلم. والإنفاق على طلبة العلم يشمل تدريبهم على المهارات الضرورية؛ لأنهم يحتاجون إليها

“ *It is legally permissible to distribute Zakat for scholarships for science passports, especially if they are in need. Even the Hanafi Madhab allows the transfer of Zakat from one country to another for distribution to seekers of knowledge. Scholarships for students include (among other things) training them in necessary skills because they are very shaky”.* ^70^

Likewise, the official fatwa institution of Jordan, namely Dairah Al-Ifta, issued a similar fatwa in 2013 in fatwa number 2847. ^71^ However, in this case, the fatwa from Dairah Al Ifta limits students’ entitlement to Zakat. The contents of the fatwa state that: 1) He must be a student who is proven to be serious about studying, so that his activities in studying cause him to be unable to work and make money. 2) The knowledge that the studies are *fardhu kifayah*, whose orientation is the good of the people, both *Shar’i* knowledge, such as *fiqh* and interpretation, and general knowledge, such as medicine, economics, chemistry, and others. 3) Zakat does not come from relatives or family obliged to provide for their needs.

From the description above, contemporary ulema are more inclined to believe that Zakat should be distributed through student scholarships. Of course, this fatwa is a breath of fresh air for the government of the Republic of Indonesia and the students themselves. For the government, this will help accelerate the human development index (HDI) ^79^ and improve the quality of human resources for the nation’s children, considering that access to higher education among young people is still very low. Alawiyah (2016) concluded that one of the leading causes is the high cost of higher education, which causes many young people of productive learning age to be unable to access it due to limited costs. ^80^ ^,^ ^81^

Education is the right of all groups, from the lower middle class to the upper middle class, with supportive socio-economic conditions; of course, there will be no difficulty in reaching education up to the upper level, but this is not the case with the lower middle class, which is why there is a need for equal distribution of opportunities to obtain education. Many weak people do not continue their education to a higher level because the cost of education is high. ^82^

### 5.3 Interpreting the Findings: Universities as Proactive Facilitators

Along with Zakat on income discourse, the findings demonstrate that universities are no longer passive recipients of Zakat allocations but have become proactive facilitators in mobilizing Zakat income to support student funding ecosystems. The convergence of fatwa-driven legitimacy, institutional innovation, and digital governance mechanisms shapes their roles. Universities are increasingly acting as collection hubs and institutionalizing Zakat governance. Universities act as micro-infrastructures for Zakat governance, transforming individual contributions into structured funding streams, like promoting auto-debit payroll systems (like in UPZ UIN Sumatera Utara), crowdfunding systems (like in PUSPAS UNAIR). By embedding Zakat collection into university financial systems, institutions improve efficiency, predictability, and *muzakki* trust.

Universities also act as boundary-spanning facilitators. Findings show that universities collaborate closely with national and private Zakat institutions to co-design scholarship schemes, like partnerships with BAZNAS and LAZISMU, that integrate university UPZ mechanisms with broader institutional frameworks. These collaborations align scholarship allocation policies with Indonesian Ulema Council fatwas, ensuring legal and theological compliance. Universities thereby bridge theological legitimacy and operational innovation, allowing contested Zakat income to be effectively mobilized for *Fisabilillah*-based scholarships.

Universities as empowerment enablers. Universities are not only managing Zakat but strategically channeling it into empowerment outcomes. Funds are allocated to support scholarships, skills development programs, and capacity-building initiatives for underprivileged students. The findings position universities as central orchestrators within Indonesia’s Zakat ecosystem. By mobilizing Zakat income, collaborating with certified Zakat bodies, and embedding digital governance, they bridge the gap between religious legitimacy and empowerment outcomes, redefining their role in higher education financing.

### 5.4 Proposed Conceptual Framework

**Figure 2.**
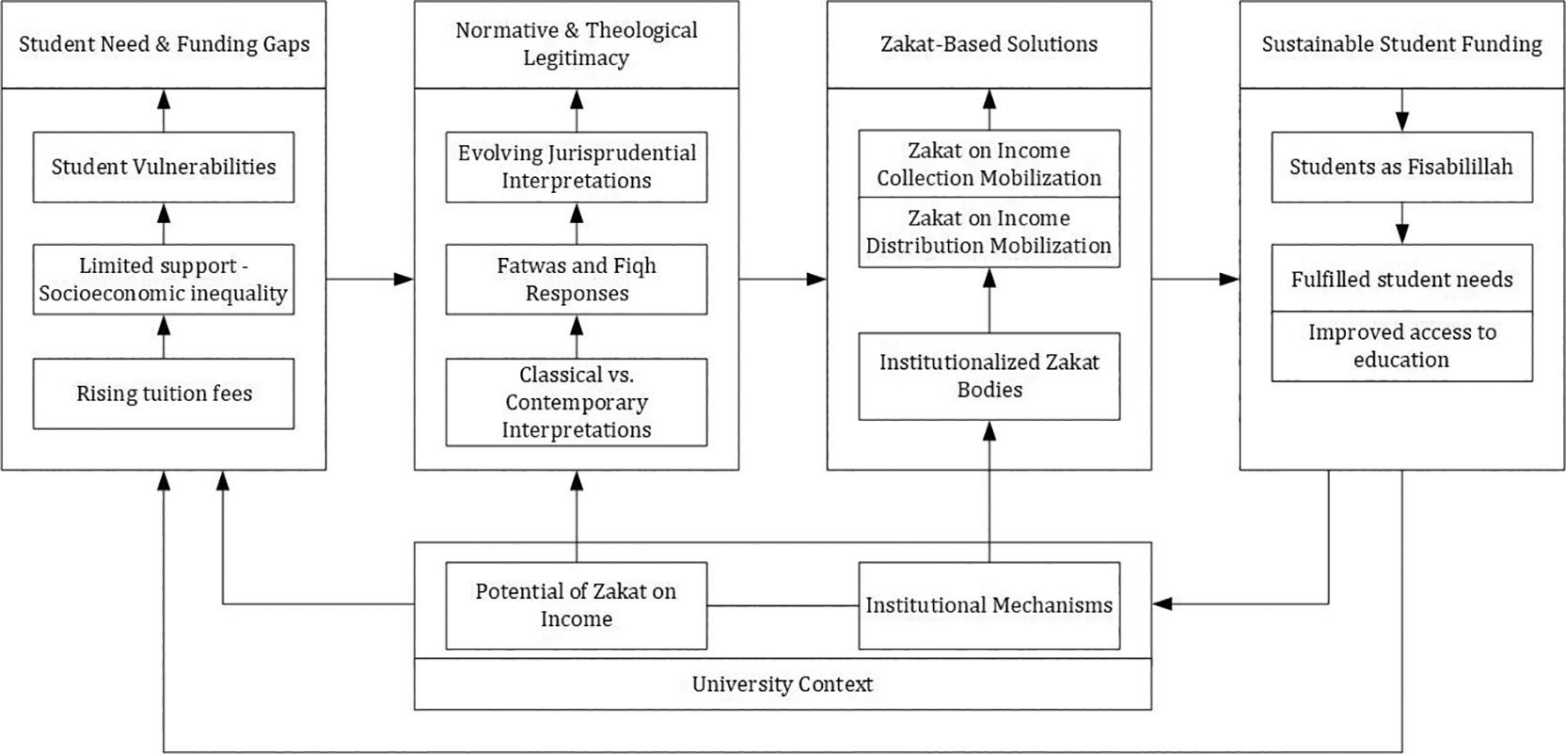
Proposed Conceptual Framework: Mobilizing Zakat on Income for Student Funding in Higher Education.

This study proposes a conceptual framework ( Figure 2) illustrating how Indonesian universities operationalize Zakat on income and *Fisabilillah*-based to address gaps in higher education funding. Fatwas issued by the Indonesian Ulema Council and other Islamic authorities provide the normative legitimacy for collecting Zakat on income and allocating funds to students under the *Fisabilillah* category. Universities leverage these fatwas as proactive facilitators by institutionalizing Zakat governance through UPZ and PUSPAS, integrating auto-debit mechanisms, and collaborating with BAZNAS and LAZISMU to mobilize resources. These funds are then distributed as scholarships, enabling broader access to education and aligning with maqasid al-shariar objectives, particularly *ḥifẓ al-‘aql* (protection of intellect) and social equity. The framework highlights an applied expansion of *fiqh*: without redefining theological foundations, Indonesian universities integrate fatwa-driven legitimacy and institutional innovation to make Zakat a sustainable funding mechanism for student empowerment.

This study reveals how Indonesian universities are reconfiguring Zakat governance to address persistent gaps in higher education funding. The findings highlight two extensions that were applied to the implementation of Zakat. First, universities mobilize Zakat on income ( *Zakatu kasbil ‘amal* ) through structured institutional mechanisms. Universities collect Zakat directly from lecturers, staff, and other stakeholders by establishing *Unit Pengumpul Zakat* (UPZ) and integrated funding centers. Leveraging Indonesian Ulema Council Fatwa No. 3/2003, universities employ digital governance systems, including auto-debit payroll mechanisms and transparent reporting dashboards, to make Zakat on income a sustainable resource for student scholarships. Second, universities utilize an inclusive interpretation of the *Fisabilillah* category as a legitimate *asnaf* for distributing Zakat-based funding solutions. While classical scholars traditionally restricted *Fisabilillah* to battle-related *jihad*, contemporary fatwas, including Indonesian Ulema Council Fatwa No. 120/1996, Dar al-Ifta Egypt (2007), and Jordan’s Fatwa No. 2847 (2013), reinterpret pursuing beneficial knowledge as a form of striving in Allah’s cause. Universities operationalize this broader *fiqh* application through structured programs like the BAZNAS Scholarship Institute.

### 5.5 Limitations, future research avenues, and implications

This study is subject to several limitations. First, it focuses on universities in Indonesia as the primary locus for examining how Zakat is mobilized to support students in need. The findings may therefore reflect context-specific dynamics, shaped by Indonesia’s strong Islamic identity and cultural traditions of mutual assistance (gotong royong). While these features make Indonesia a fertile ground for integrating Zakat into higher education funding, they may limit the generalizability of the findings to other contexts with different socio-religious environments.

Future studies could explore broader models of Islamic-based social finance in higher education beyond Zakat: *Alms* (infaq) and voluntary giving to investigate how these mechanisms can complement Zakat in supporting students. Waqf-based funding models, to initial observations, indicate that UNIDA Gontor and several other Indonesian universities have successfully integrated endowment-based financing into their governance. Comparative studies into cross-country analyses could reveal how different cultural, legal, and institutional frameworks shape the mobilization of Islamic social funds in higher education.

The findings have significant implications for universities, Zakat institutions, and policymakers. For Universities, the study demonstrates the potential of establishing *Unit Pengumpul Zakat* (UPZ) or dedicated funding bodies, such as a private Zakat institution, to mobilize Zakat income effectively. Integrating Zakat into institutional strategies can enhance scholarship provision and ensure broader educational access. Collaboration with universities can strengthen Zakat institutions’ governance, making distribution more targeted and aligned with academic needs. For Policymakers, insights from this study may inform frameworks for integrating Islamic-based social funds into national education financing strategies. By aligning fatwa-driven legitimacy with institutional innovation, Indonesian universities have successfully leveraged Zakat to support underprivileged students, providing a practical model that can inspire higher education funding strategies elsewhere.

## Data availability

All data underlying the results is available as part of the article and no additional source data are required.
