# Supplementary material for: Distributing Zakatu Kasbil 'Amal as an Alternative to Student Funding, Evidence in Indonesia’s Universities
Source: F1000Res. 2025 Sep 22;13:185. Originally published 2024 Mar 11. [Version 2] doi: 10.12688/f1000research.144610.2 (PMC12583907; doi:10.12688/f1000research.144610.2)
Supplement: Peer review history

# Reviewer report: Reviewer response for version 2

**DOI:** 10.5256/f1000research.188022.r418974

**Published:** 2025-11-03

**Authors:** Jonathan O'Donnell

**Affiliations:** 1 Deakin University, Burwood, Victoria, Australia

**Where I stand**

 Before I begin, I should say that I am not an expert in Muslim funding mechanisms, nor am I an expert in student funding in Indonesian universities. 

 My expertise sits with alternative funding sources for universities. I have particular expertise in crowdfunding for research at Australian universities. 

 My review should be read in this light - as an outsider to the topic, who sits within the broader field of alternative funding mechanisms. 

**Article strengths**

 This article is a useful review of the current state of Zakat funding for student scholarships at Indonesian universities. It's value comes from it's focus on this type of funding, which is not well understood outside of the Muslim world, and its focus on Indonesian universities. 

 I complement the authors on the conceptual focus of the article. The issue of funding higher education is a worldwide debate, with many different funding models in different jurisdictions. By restricting this article to focus on Zakat as a funding mechanism, within universities in the Indonesian jurisdiction, they are able to focus clearly on this particular model. 

 The article gains its strength from combining academic literature, institutional documentation and legal rulings. 

 The narrative thread through the document is clear and lead the reader to the conclusions. 

**Improvements**

 I think that the work done on the literature could be clarified by clearly stating how many documents were reviewed in each category. The text mentions "28 studies reviewed (2003 - 2024)", but it isn't clear whether that is 28 documents including all academic articles, institutional documentation and fatwas, or whether it is 28 academic articles. The authors have clearly done a lot of work, and that work should be reflected in a table or text that clarifies how many documents in each category were considered. 

 The dates also should be aligned: 

 * In Table 2, the inclusion criteria states: "Published after 2010."

 * In the text it says "...28 studies reviewed (2003–2024)...". 

 Are the articles only from 2010 onwards, or are they from 2003 onwards? 

 Are these two statements about different types of documents, with the articles from 2010 and the institutional documentation and fatwas from 2003? 

 As I said before, a table or text that clarifies how many documents of each type. If it included the date ranges for earliest and latest document considered for each of the three types, that would be useful for the reader to understand the scope of the review. 

 In overall terms, it isn't clear to me whether Zakat for scholarships is a common practice across all Indonesian universities (and therefore this paper is trying to regularise that practice) or Zakat for scholarships is an emerging practice across all Indonesian universities (and therefore this paper is trying to understand and perhaps encourage that practice). It is beyond the scope of the article to say how many universities are doing this, and how many students benefit, but it would be good to provide a general sense of whether the four examples listed in Table 4 are good examples drawn from all universities, or are some of the few examples that exist today. This would give readers from outside of Indonesia a sense of the scale of the practice at the time of writing. 

**Minor issues**

 Section 4.2 and 4.3 have the same heading text. 

 There are some words capitalised that are not proper nouns.

Are the rationale for, and objectives of, the Systematic Review clearly stated?

Yes

Is the statistical analysis and its interpretation appropriate?

Not applicable

If this is a Living Systematic Review, is the ‘living’ method appropriate and is the search schedule clearly defined and justified? (‘Living Systematic Review’ or a variation of this term should be included in the title.)

Not applicable

Are sufficient details of the methods and analysis provided to allow replication by others?

Partly

Are the conclusions drawn adequately supported by the results presented in the review?

Yes

Reviewer Expertise:

My expertise connects with this paper in the broad area of alternative methods of funding in universities. My expertise differs from the authors in that I focus on research funding in Australian universities.

I confirm that I have read this submission and believe that I have an appropriate level of expertise to confirm that it is of an acceptable scientific standard.

# Reviewer report: Reviewer response for version 2

**DOI:** 10.5256/f1000research.188022.r416421

**Published:** 2025-09-30

**Authors:** Ade Nur Rohim

**Affiliations:** 1 Universitas Pembangunan Nasional Veteran Jakarta, Jakarta, Indonesia

The article has undergone substantial improvements and now provides a comprehensive overview of the zakat management model aimed at enhancing the education sector.

Are the rationale for, and objectives of, the Systematic Review clearly stated?

Partly

Is the statistical analysis and its interpretation appropriate?

Not applicable

If this is a Living Systematic Review, is the ‘living’ method appropriate and is the search schedule clearly defined and justified? (‘Living Systematic Review’ or a variation of this term should be included in the title.)

Not applicable

Are sufficient details of the methods and analysis provided to allow replication by others?

No

Are the conclusions drawn adequately supported by the results presented in the review?

No

Reviewer Expertise:

Islamic Economics; Zakat; Waqf

I confirm that I have read this submission and believe that I have an appropriate level of expertise to confirm that it is of an acceptable scientific standard.

# Reviewer report: Reviewer response for version 2

**DOI:** 10.5256/f1000research.188022.r416422

**Published:** 2025-09-26

**Authors:** Muhammad Irwan Ariffin

**Affiliations:** 1 International Islamic University Malaysia, Kuala Lumpur, Malaysia

1. Introduction: Paragraph 4 - the authors started by highlighting the controversy on zakat on income (zakat kasb amal) but went on discussing the zakat of scholarship under fi sabilillah asnaf - these two are totally different issues. If Indonesia follows Mazhab Shafie, then there must be an explanation why and how zakat on income is accepted since it is rejected in Mazhab Shafie. 

 2. Section 2.1 Explain how zakat on income (zakat kasb amal) was established and practiced in Indonesia.

 3. Section 2.4 - The structure of institutionalised zakat bodies in universities is not clear - are they part of the university management structure, who do they report to, how to maintain good governance?

 4. There were some repetitions on the issue of zakat fi sabilillah extended to scholarship in different places throughout the article.

 5. From the first round of review, I asked for some data on zakat kasb amal collection, zakat fi sabilillah disbursed in Indonesia, etc. No data available?

 6. Please send the article to a proofreader. The writing style, spelling and grammar need to be improved.

Are the rationale for, and objectives of, the Systematic Review clearly stated?

Yes

Is the statistical analysis and its interpretation appropriate?

No

If this is a Living Systematic Review, is the ‘living’ method appropriate and is the search schedule clearly defined and justified? (‘Living Systematic Review’ or a variation of this term should be included in the title.)

Partly

Are sufficient details of the methods and analysis provided to allow replication by others?

Partly

Are the conclusions drawn adequately supported by the results presented in the review?

No

Reviewer Expertise:

Applied Macroeconomics, Theoretical Microeconomics, Islamic Economics, Social Finance, Public Finance.

I confirm that I have read this submission and believe that I have an appropriate level of expertise to confirm that it is of an acceptable scientific standard, however I have significant reservations, as outlined above.

# Reviewer report: Reviewer response for version 1

**DOI:** 10.5256/f1000research.158428.r400985

**Published:** 2025-08-25

**Authors:** Muhammad Irwan Ariffin

**Affiliations:** 1 International Islamic University Malaysia, Kuala Lumpur, Malaysia

1. The title does not truly reflect the content of the article - while there are discussions on the origin of zakat on income and students as fi sabilillah asnaf in INDONESIA, there is a serious lacking of link with Indonesia's universities. Some of the keywords are also not properly discussed - improving university quality, indonesian universities, and student funding. 

 2. If the title is to be maintained - then they must be a stronger link with Indonesian universities - for example, how many indonesian university students received or benefited from zakat under fi sabilillah, did the quality of indonesian universities improved after their students received zakat under fi sabilillah (but this is difficult to prove since the students are the one that receive zakat, not the university management), etc.

 3. The use of social funds by universities' philanthropic organizations is different than zakat - since social funds can be channeled directly to university management for development and operational expenses - but zakat must be given to students. So the empirical data collected for this study seems not really aligned with the objective/title.

 4. Suggest to provide data on income zakat and zakat for students in Indonesia to motivate the research. Did zakat for students increases after zakat on income has become popular due to the translation of Qaradhawi's Fiqh Zakat in Indonesia? Or, is the research actually focusing on income zakat collection from university's staff which are then channeled to their students under fi sabilillah? Is this the model that the research is trying to analyze/develop?

 5. Suggest for the article to be sent for professional proofreading service to improve the language style and enforce consistency in spelling - MUI or Indonesian Ulema Council, Al Quran or Koran, Fisabilillah (capital F) or fisabilillah? Madhab or Madhhab/Mazhab? Is it correct to call Islamic State of Jordan?

Are the rationale for, and objectives of, the Systematic Review clearly stated?

Yes

Is the statistical analysis and its interpretation appropriate?

No

If this is a Living Systematic Review, is the ‘living’ method appropriate and is the search schedule clearly defined and justified? (‘Living Systematic Review’ or a variation of this term should be included in the title.)

Partly

Are sufficient details of the methods and analysis provided to allow replication by others?

Partly

Are the conclusions drawn adequately supported by the results presented in the review?

No

Reviewer Expertise:

Applied Macroeconomics, Theoretical Microeconomics, Islamic Economics, Social Finance, Public Finance.

I confirm that I have read this submission and believe that I have an appropriate level of expertise to state that I do not consider it to be of an acceptable scientific standard, for reasons outlined above.

# Response

**Published:** 2025-09-11

**Authors:** Budi Dharma

Dear Muhammad Irwan Ariffin.

 with respect.

 We sincerely thank the reviewer for the constructive feedback. We have carefully revised the manuscript to address all comments point by point:

**1. Title and Content Alignment.**

 We decided to maintain the current title but have strengthened the manuscript to better support it. Specifically, we:

- Expanded discussions on how Indonesian universities integrate Zakat into student funding strategies.
- Added contextual data showing how Zakat on income *(zakatu kasbil ‘amal) *is institutionalized through UPZs, private Zakat institution, or university partnerships with BAZNAS and LAZISMU.
- Included examples of structured scholarship programs that operationalize the *Fisabilillah *category within universities.

2. **Stronger Link with Indonesian Universities**

 We have improved the connection between Zakat and higher education by:

- Adding **national data** on zakat-based scholarships. For example, BAZNAS Scholarship Institute supported **1,670 students** in 2023, while LAZISMU allocated **IDR 6.6 billion** to fund **6,600 students** in 2021.
- Including **institutional case studies** such as UNAIR’s PUSPAS, UIN Sumatera Utara to demonstrate the operational models.
- Clarifying universities role as proactive facilitators, by Institutionalized Zakat bodies in Universities, facilitated the Zakat collection and distribution.
- Clarifying that Zakat’s role is reducing financial exclusion for students.

**3. Clarification of Zakat vs. Social Funds**

 We have **clarified in the discussion** that zakat distribution must comply with Islamic legal principles, particularly *tamalluk* (direct ownership), meaning that zakat funds are allocated **directly to eligible students** under the *Fisabilillah* category rather than being used for university operational expenses. Universities act as facilitators that support zakat collection and scholarship programs, rather than as recipients of zakat themselves. here are the example of quote:

 “Table 4 summarizes how universities and Zakat institutions operationalize Zakat income to support student scholarships under the *fisabilillah* category”.

**4. Data on Income Zakat and Student Funding Models**

 To strengthen the empirical foundation, we:

- Added **historical context** on the increasing acceptance of zakat on income following Qaradawi’s *Fiqh al-Zakat*.
- Highlighted how **income zakat collected from university staff** is redistributed to students under the *Fisabilillah* category.
- Summarized these practices in **Table 4**, showing zakat collections, distribution mechanisms, and the number of students funded.

**5. Language and Terminology**

 We have conducted thorough proofreading and ensured consistent terminology across the manuscript:

- “MUI” standardized into "Indonesian Ulema Council"
- “Al-Qur’an” used consistently
- “Fisabilillah” consistently capitalized and Italic

We believe these revisions make the manuscript clearer, more focused, and better aligned with the title and research objectives. We are grateful for the reviewer’s comments, which have significantly strengthened the work.

 Warms Regards.

# Reviewer report: Reviewer response for version 1

**DOI:** 10.5256/f1000research.158428.r355629

**Published:** 2025-01-17

**Authors:** Ade Nur Rohim

**Affiliations:** 1 Universitas Pembangunan Nasional Veteran Jakarta, Jakarta, Indonesia

This research is quite relevant to the current phenomenon of zakat distribution which has been widely implemented in various zakat institutions.

 However, some notes for the author to consider include:

 1. The abstract does not contain a sufficient statement of the research problem, especially related to the distribution of zakat to the scholarship scheme. This has implications for the bias of the approach used, whether using a fatwa approach, or regulation, or an interpretation (tafseer) approach or indeed only limited to a review of several of these provisions.

 2. There are terms in the abstract that trigger fundamental errors, such as nisab which is interpreted as "1 year", and also the number of asnaf mentioned as 9 asnaf. The use of the term "Baznas" also needs to comply with the provisions of the Zakat Law which no longer uses the term "BAZ".

 3. The research results presented in the abstract are not relevant enough to the discussion. The author states the practice in "several universities" related to zakat distribution, but this is not specifically reviewed in the discussion, including its impact on the success of student studies which has not been described in depth.

 4. Reviews of several previous studies related to the research topic have not been clearly stated in the introduction. The author needs to describe them to strengthen the justification of the novelty of this research, including by describing academic debates related to this research topic.

 5. There needs to be a comprehensive explanation regarding the terms used, whether zakat kasbil 'amal or income zakat, by reviewing the similarities or differences, or the relationship between the two terms.

 6. The author needs to review several practices of implementing zakat distribution at several universities in Indonesia in a separate subsection in the discussion section, so that it can be relevant to the title of this research which captures a portrait of zakat distribution at various universities in Indonesia.

 7. There is no final conclusion to answer the research questions.

 Based on these notes, this study is expected to reconstruct the systematic discussion in this study to make it in line between the research title, methods and approaches used, and the findings and results of the study that answer the research questions.

Are the rationale for, and objectives of, the Systematic Review clearly stated?

Partly

Is the statistical analysis and its interpretation appropriate?

Not applicable

If this is a Living Systematic Review, is the ‘living’ method appropriate and is the search schedule clearly defined and justified? (‘Living Systematic Review’ or a variation of this term should be included in the title.)

Not applicable

Are sufficient details of the methods and analysis provided to allow replication by others?

No

Are the conclusions drawn adequately supported by the results presented in the review?

No

Reviewer Expertise:

Islamic Economics; Zakat; Waqf

I confirm that I have read this submission and believe that I have an appropriate level of expertise to confirm that it is of an acceptable scientific standard, however I have significant reservations, as outlined above.

# Response

**Published:** 2025-09-11

**Authors:** Budi Dharma

Dear Ade Nur Rohim,

 with respect,

 We thank the reviewer for the constructive and detailed feedback. We have carefully revised the manuscript to address each point and improve the overall clarity, consistency, and alignment between the title, methodology, and findings. Our responses are as follows:

**1. Abstract and Research Problem Clarification**

 We have **revised the abstract** to explicitly state the research problem and clarify the scope of our inquiry. The abstract now explains the funding gap in Indonesian higher education, highlights the growing use of zakat on income ( *zakatu kasbil ‘amal*) for scholarships, and describes how this study investigates institutional practices and theological frameworks that enable zakat distribution for students under the *Fisabilillah* category.

**2. Terminology Accuracy and Consistency**

*Comment:* Some terms in the abstract were inaccurate, such as interpreting *nisab* as “1 year,” miscounting asnaf as “9,” and inconsistent use of “Baznas.”

*Response:* We have **corrected all terminology** to ensure consistency and compliance with zakat regulations:

- Clarified that *nisab* refers to the **minimum wealth threshold**, not duration.
- Confirmed there are **eight asnaf categories**, in line with QS. At-Taubah: 60.
- Standardized the use of **BAZNAS** (Badan Amil Zakat Nasional), aligning with the Zakat Law terminology.

**3. Linking Abstract Results to the Discussion**

 We have strengthened the discussion by explicitly integrating **case-based examples** from several Indonesian universities:

- **PUSPAS UNAIR** — integrates zakat and sadaqah into structured tuition support.
- **UPZ UIN Sumatera Utara** — mobilizes payroll-based zakat and distributes scholarships.
- We also highlight the role of the **BAZNAS Scholarship Institute** and **LAZISMU**, ensuring consistency between the abstract and the discussion. 

**4. Review of Related Studies and Theoretical Debates**

 We have expanded the introduction to include **key prior studies and scholarly debates** on:

- The theological controversies around using zakat for education ( *fisabilillah* interpretation).
- Institutional innovations enabling university-based zakat management.
- Empirical trends showing the increasing use of zakat for student funding.
  This positions our research within ongoing debates and clarifies its **novel contribution**.

**5. Clarification of Zakat Kasbil ‘Amal vs. Income Zakat**

 We have added a clarification in the literature review, noting that **zakat kasbil ‘amal** is the **Arabic term** for zakat on income derived from professional work or salaries, while “income zakat” is its direct English equivalent. Both terms are now used interchangeably but consistently, and this clarification resolves potential ambiguity.

**6. Institutional Practices of Zakat Distribution**

 We created a distinct subsection within the discussion presenting **institutional practices of zakat-based scholarships**, including:

- **UPZ mechanisms** at UIN Sumatera Utara.
- **Integrated models** like PUSPAS UNAIR combining zakat, infaq, and sadaqah.
- **National and private initiatives** like the BAZNAS Scholarship Institute and LAZISMU’s *Beasiswa Sang Surya*.
  This makes the discussion **directly aligned** with the research title.

**7. Strengthened Conclusion**

 We have rewritten the conclusion to **directly answer the research questions** by:

- Summarizing how zakat on income has been institutionalized within Indonesian universities.
- Highlighting how fatwa-driven legitimacy enables broader use of the *Fisabilillah* category.
- Emphasizing universities’ evolving roles as proactive facilitators of zakat-based student funding.

**8. Alignment Between Title, Methods, and Findings**

 We have restructured the manuscript to **improve the flow and alignment** between the title, methodology, and findings:

- The **methods** now clearly explain how the systematic review integrates theological, regulatory, and institutional perspectives.
- The **discussion** explicitly links findings to institutional case studies.
- The **conclusion** ties back to the research objectives and answers the central questions.

We believe these revisions have substantially improved the manuscript’s clarity, depth, and alignment. We thank the reviewer for the valuable insights, which have significantly strengthened our work.

 Warm Regards.
